# Singular manifolds of proteomic drivers to model the evolution of inflammatory bowel disease status

**DOI:** 10.1038/s41598-020-76011-7

**Published:** 2020-11-04

**Authors:** Ian Morilla, Thibaut Léger, Assiya Marah, Isabelle Pic, Hatem Zaag, Eric Ogier-Denis

**Affiliations:** 1 Université Sorbonne Paris Nord, LAGA, CNRS, UMR 7539, Laboratoire d’excellence Inflamex, F-93430 Villetaneuse, France; 2 INSERM, Research Centre of Inflammation, Laboratoire d’excellence Inflamex, BP 416, Paris, France; 3 UMR 7592 CNRS, Institut Jacques Monod, Université Paris Diderot, Paris, France; 4 Université Rennes, Inserm, EHESP, Irset - UMRS 1085, 35000 Rennes, France; 5 Inception IBD Inc., Montreal, Canada

**Keywords:** Biophysics, Computational biology and bioinformatics, Drug discovery, Immunology, Molecular biology, Systems biology, Biomarkers, Gastroenterology, Molecular medicine

## Abstract

The conditions used to describe the presence of an immune disease are often represented by interaction graphs. These informative, but intricate structures are susceptible to perturbations at different levels. The mode in which that perturbation occurs is still of utmost importance in areas such as cell reprogramming and therapeutics models. In this sense, module identification can be useful to well characterise the global graph architecture. To help us with this identification, we perform topological overlap-related measures. Thanks to these measures, the location of highly disease-specific module regulators is possible. Such regulators can perturb other nodes, potentially causing the entire system to change behaviour or collapse. We provide a geometric framework explaining such situations in the context of inflammatory bowel diseases (IBD). IBD are severe chronic disorders of the gastrointestinal tract whose incidence is dramatically increasing worldwide. Our approach models different IBD status as Riemannian manifolds defined by the graph Laplacian of two high throughput proteome screenings. It also identifies module regulators as singularities within the manifolds (the so-called singular manifolds). Furthermore, it reinterprets the characteristic nonlinear dynamics of IBD as compensatory responses to perturbations on those singularities. Then, particular reconfigurations of the immune system could make the disease status move towards an innocuous target state.

## Introduction

The way a living system responds to threats is a decision-making process depending on multiple factors. Some of those factors such as limited resources or energetic cost shape the different phenotypic status of a disease. Hence, the identification of “transition gates” (hereinafter referred as singularities) between those phenotypic phases opens a path to eventual therapeutic interventions that reconfigure the system to a non inflamed status. In particular, these status can be expressed by different grades of inflammation when the immune system of the gastrointestinal tract reacts to, for instance, the presence of harmful stimuli or simply to a drop in commensal microbiota tolerance. If the inflammatory conditions of the colon and small intestine become chronic, then, they are all generally grouped under the heading of Inflammatory bowel disease (IBD). IBD is an intestinal disease of unknown cause whose prevalence is in continuous growth at present. Crohn’s disease (CD) and ulcerative colitis (UC) are the main sub-types of IBD. UC, for instance, is characterised by chronic inflammation and ulceration of the lining of the major portion of the large intestine (colon). Unlike CD, the most of the patients are diagnosed later in life^1,2^ and according to the European Medicines Agency presents a prevalence of 24.3 per 100, 000 person-years in Europe. That means there are between 2.5 and 3 million people who have IBD in the European Union^3^ and this figure could be increased to 10 million worldwide in 10 years^4^. Thus, the particular effect of UC on health-care systems will be exponentially growing since its incidence continues to rise with 178, 000 new cases of UC each year. In addition, IBD is a continuous chronic pathological state that may produce aberrant cell proliferation leading to broad epithelial alterations such as dysplasia. This scenario of chronic active inflammation in patients with UC increases the risk for the development of colorectal carcinoma (CRC) and often requires total colectomy in case of intensive medical treatment failure or presence by high-grade dysplasia. Thus, detecting and eliminating or even reverting precursor dysplastic lesions in IBD is a practical approach to prevent the development of invasive adenocarcinoma. In such cases, practitioners have to make a decision on the new therapy to use only based on their grade of expertise in inflammatory domains. The limited and largely subjective knowledge on this pathology encouraged us to seek biomarker(s) whose symbiotic actions influence the molecular pathogenesis of the risk for colorectal cancer in IBD. In this work, we abstract the IBD progression by means of manifolds created from protein expression profiles. Thus, we can assamble a dynamic framework where to identify key proteins prior to develop dysplasia. Since the IBD behaviour can be naturally observed under the prism of a “phase transition” process^5^, we sample the expression profiles of patients from a manifold with singularities; evaluating the functions of interest to the IBD status geometry near these points. Yet in the mucosa, various proteomic signatures have been identified in both active and inactive patients of IBD^6^. Thus, we hypothesised that protein biomarkers could help to predict the therapeutic response in patients with different status of the disease progression. Additionally, we explored other possible connections with biomarkers of dysplasia. Given this assumption, we construct weighted protein co-expression graphs of each disease sub-type by means of a proteomic high-throughput screening consisting of two cohort of 20 patients each (replica 1 and replica 2). Next, we localise proteins regulating any module identified by Weighted Gene co-Expression Network Analysis (WGCNA)^7^ as relevant to the IBD status, i.e., control, active and quiescent. Then, we use functions associated to the eigengenes of selected proteins across patients^7^ to describe the potential of protein expressions with respect to the disease status. And finally, we lay emphasis on the behaviour of the graph Laplacians corresponding to points at or near singularities, where different transitions of disease come together. This scenario enables the identification of potential drug targets in a protein-coexpression graph of IBD, accounts for the nonlinear dynamics inherent to IBD evolution and opens the door to its eventual regression to a controlled trajectory^8–10^. Overall, this manuscript envisages providing clinicians with useful molecular hypotheses of disease activity status prior to making any decision on the newest course of the treatment of individual patients in IBD. In line with this, our systemic approach could ultimately facilitate and accelerate drug discovery in health-care system.

## Results

### WGCNA identifies novel immune drivers causing singularities in the status of disease progression

**Figure 1:**
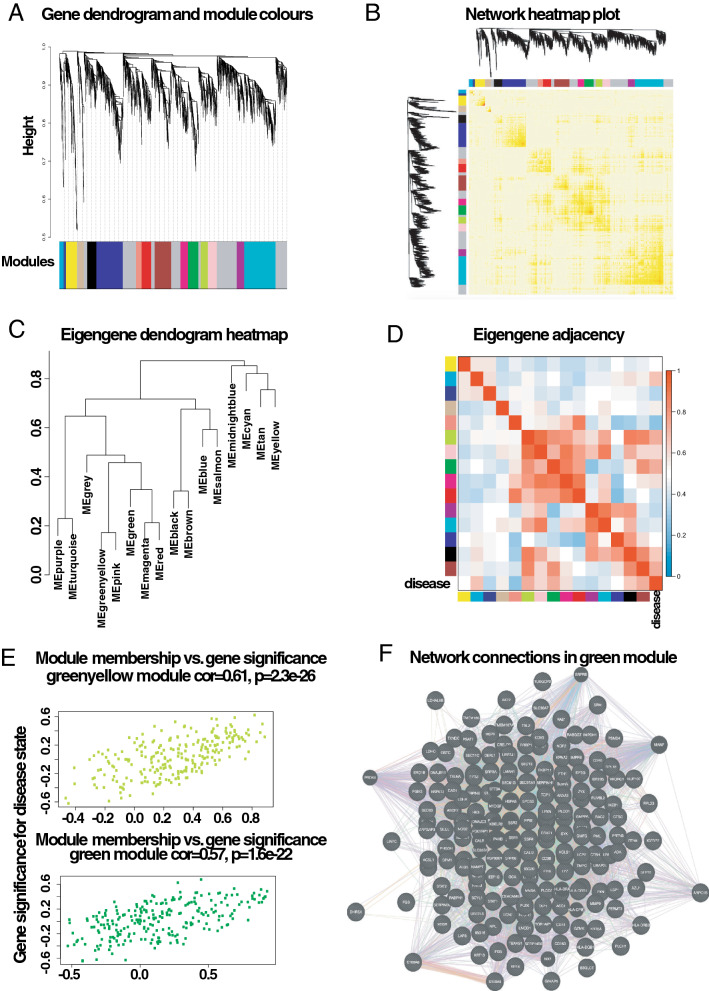
Overview of the protein coexpression network analysis in UC. (**A**) Hierarchical cluster tree of the 3910 proteins analysed. The colour strips simply display a comparative overview of module assignments by means of a method that cuts the branches dynamically as introduced in^15^. Modules in grey are composed by “housekeeping” proteins. (**B**) Topological Overlap Matrix (TOM) plot (also known as connectivity plot) of the network connections. We rank the proteins in the rows and columns following the clustering tree classification. The colour scheme smoothly ranges from faint towards thick nuances according to a lower or a higher topological overlap. Typically data clusters along the diagonal. We also include both the cluster tree and module assignment that lie on the left and top sides respectively. (**C**) Hierarchical clustering dendrogram of the eigengenes calculated by the dissimilarity measure $diss(q_{1},q_{2})=1-cor(E^{(q_1)},E^{(q_2)}$)^15^. (**D**) Eigengene network visualisation that amounts to the relationships among the modules and the disease status. The eigengene adjacency $A_{q_{1,}q_{2}}=0.5+0.5cor(E^{(q_{1})},E^{(q_{2})}$)^15^. (**E**) Protein significance (*PS*) versus module membership (*MM*) for disease status related modules. Both measurements keep a high correlation enhancing the strong interrelations between the IBD progression and the respective eigengenes module (i.e. greenyellow and green). $MM^{mod.colour}(i)=cor(x_{i},E^{mod.colour})$^15^. (**F**) The green module graph enriched with subgraphs functionally involved in IBD progression.

**Figure 2:**
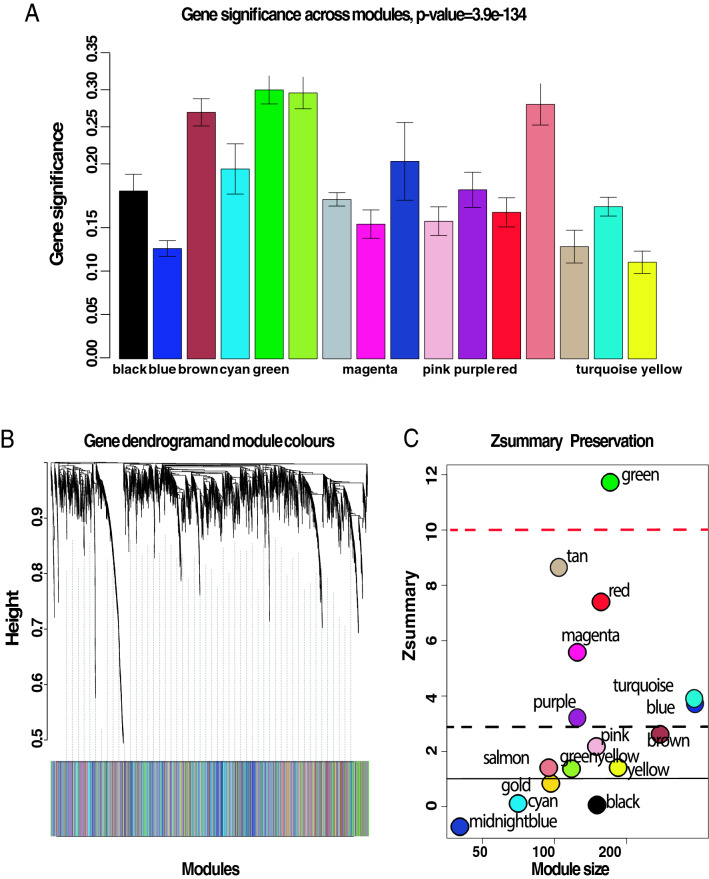
Significance and preservation of UC graph modules. (**A**) The module significance (protein significance in average) of the modules. The underlying protein significance is defined with respect to the patient disease status. (**B**) The consensus dendrogram for replica 1 and replica 2 of the UC co-expression graphs. (**C**) The composite statistic *Zsummary* (Eq.9.1 in^15^). If $Zsummary>10$ the probability the module is preserved is high^16^. If $Zsummary<2$, we can say nothing about the module preservation. In the light of the Zsummary, it is apparent there exists a high correlation with the module size. The green UC module shows high evidence of preservation in its two replica graphs.

**Figure 3:**
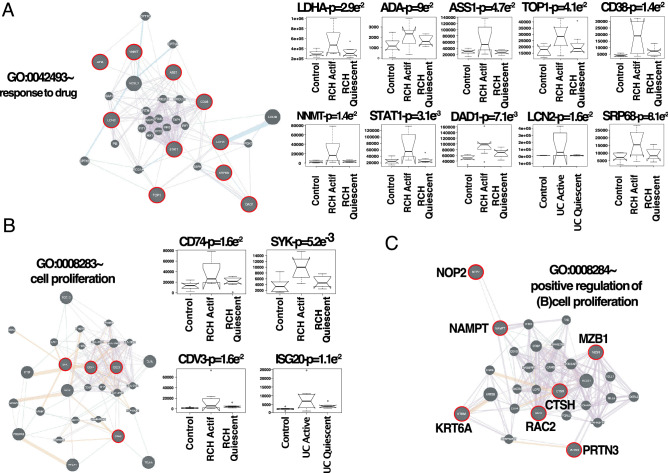
Representative repertoire of graphs by enriched functions that are well preserved in UC. (**A**) Response to drug. (**B**) Cell proliferation. (**C**) Positive regulation of B cell proliferation. Edge colouring in graphs: purple stands for Co-expression, orange for Predicted, light-blue for Pathway, light-red for Physical interactions, green for Shared protein domains and blue for Co-localisation. Boxplots of expression for the most attractive drivers respect to UC status are indicated by red circles at the top of each subgraph. Initial P upon the boxplot title amount to p-value associated with the IBD status correlation, whereas PC are the initials to positive control, i.e., proteins already described as IBD-related. For the sake of simplicity, we highlighted some candidates simply by its identifiers. See Fig. S0 for more details on networks.

**Figure 4:**
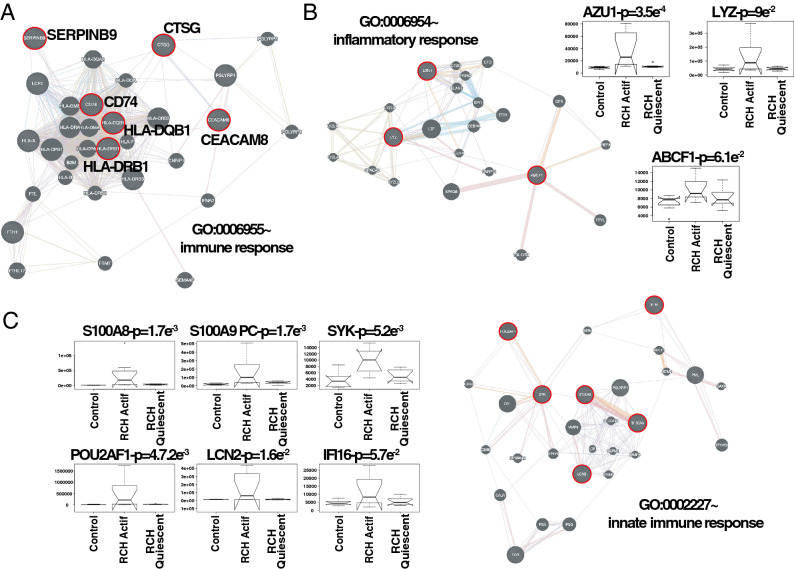
Representative repertoire of graphs by enriched functions that are well preserved in UC. (**A**) Immune response. (**B**) Inflammatory response. (**C**) Innate immune response. The protein interaction graphs were constructed using Genemania^17^. Edge colouring in graphs: purple stands for Co-expression, orange for Predicted, light-blue for Pathway, light-red for Physical interactions, green for Shared protein domains and blue for Co-localisation. Boxplots of expression for the most attractive drivers respect to UC status are indicated by red circles at the top of each subgraph. Initial P upon the boxplot title amount to p-value associated with the IBD status correlation, whereas PC are the initials to positive control, i.e., proteins already described as IBD-related. For the sake of simplicity, we highlighted some candidates simply by its identifiers. See Fig. S0 for more details on networks.

Intuitively, one might envisage the progression of a disease as a set of immune subsystems influencing each other as response to an undesired perturbation of the normal status. In this exchange, there exist specific configurations that cause the entire system to change its behaviour or collapse. We were, then, interested in identifying the potential modulators of IBD state whose interactions may explain the disease progression as a system instead of simply investigating disconnected drivers dysregulated in their expression levels. To this end, we provide significance measures (*PS*) of protein co-expression graphs to each type of the disease (CD and UC). Conveniently to our purposes, those measures are simultaneously topological and biologically meaningful since they are defined by $cor|x_{i},S|^{\xi }$ with $\xi \ge 1$ and are based on the clinical outcomes of two proteomic samples (SI Text) capturing the IBD phenotype or status, i.e., control, active and quiescent. We fixed this status as a quantitative trait defined by the vector $S = (0,1,-1)$. And applied the Weighted Gene co-Expression Network Analysis^11,12^ between the two samples, herein considered as replica 1 and replica 2 cohorts. For the sake of clarity, we only show the results obtained for the UC graph (Fig. 1). The matched inspection of its expression patterns in connectivity (Fig. 1A), hierarchical clustering of their eigengenes^13^ and its eigengene adjacency heatmap (Fig. 1B–D) suggests that the most correlated expression patterns with the IBD status (Figs. 1E and 2A) are highlighted in greenyellow (97 proteins) and green (215 proteins) respectively. Whereas in CD those coloured in magenta (123 proteins) and midnightblue (41 proteins) yielded the highest correlations (Fig. S1). Nevertheless, we only kept green (UC) and magenta (CD) patterns since the others were not well preserved in the graph corresponding to the validation cohort (Fig. 2B,C and Fig. S2). Those two modules exhibited an intersection of about the $14\%$ (Table S3). Next, we wonder about the biological functions these patterns of similar protein expression to the IBD status were enrich of. To response this question, the weighted co-expression subgraph of the green (resp magenta) pattern was interrogated (Fig. 1F) using GO^14^. As we expected, the green and magenta expression patterns present overabundance of IBD-related with multiple processes that are essential for the disease progression (Tables 1 and S1) such as positive regulation of B cell proliferation (Fig. 3C), inflammatory response or innate immune response in mucosa (Fig. 4B,C). Complementary, pathways highly related to the disease progression such as intestinal immune network for IgA production (hsa04672)^6^ or known pathways such Inflammatory bowel disease (hsa:05321) are also found (Table S2). Some surprising terms, especially other diseases such as tuberculosis, influenza A, and diabetes, appeared more enriched than IBD maybe suggesting commun signalling pathways. In addition, the intersection of dysregulated protein sets involved in such pathways between UC and CD is very low (Figs.3, 4 and Fig. S3). As positive control the differential expression of well-known proteins participating in IBD such as CAMP or LYZ in UC and LCN2 or IFI16 both in UC and CD are also detected. In particular, the set composed by the proteins STAT1, AZU1, CD38 or NNMT in UC or DEFA1, IGHM, PGLYRP1 and ERAP2 in CD are robustly associated with the status of the disease (see Tables 1 and S2). These nodes, and other frequently-occurring nodes such as SYK and CD74, are attractive candidates for experimental verification. Some of these proteins work in tandem, with control sets formed by S100A9 and S100A8 identified in innate immune response process. Strikingly, some proteins such as CTSH presented in processes such as adaptive immune response or regulation of cell proliferation show similar expression between active and quiescent UC status (Fig. S4). Moreover, in CD the regulation of immune response process displayed similar expression in the active and quiescent status for all its proteins mostly belonging to the immunoglobulin heavy variable protein family that participates in the antigen recognition (Fig. S5). It is also worth mentioning that those proteins more expressed in the quiescent than in the active status such as SYK or IGKV2-30 are mainly identified during the CD progression (Fig. S6). Upon the performance of this functional analysis, a total of 37 WGCNA candidate proteins were selected as disease-relevant within the green module of UC. Whereas the selected proteins in the case of the magenta module in CD were 19 (SI text).

**Table 1:** Analysis of the biological functions enriched in GO. We provide the proteins associated with the green module inferred by the WGCNA UC analysis along their multi-test corrected p-values.

Biological process in GO	Proteins	Green module
Immune response	HLA-DQB1, CD74, CEACAM8, SERPINB9, IGLV3-5, IGHV3-53	$p=1.3e^{-5}$
Response to drug	LDH, NNMT, ADA, STAT1, ASSQ, DAD1, LCN2, CD38, SRP68	$p=2e^{-3}$
Innate immune response in mucosa	S100A9, DEFA1, S100A8, SYK, IFI16	$p=6e^{-3}$
Adaptive immune response	CTSH, TAP1	$p=4e^{-2}$
+ Regulation of cell proliferation	KRT6A, NOP2, CTSH, CAMP, RAC2, MZB1, PRTN3, NAMPT	$p=7e^{-2}$
Cell proliferation	CD74, REG1B, CDV3, ISG20	$p=1.6e^{-2}$
Inflammatory response	AZU1, LYZ, ABCF1	$p=8e^{-3}$

In the light of these computational predictions, we hypothesise the effectiveness of dynamically addressing short-term actions on the identified novel immune drivers in changing the IBD status. Note that the identification of such modifications from scratch by means of purely experimental screenings might be not tractable.

**Table 2:** Manifolds incidence $\theta$, kernel search $\phi _{\kappa }$ and eigengene form $f_{i}(x)$. First row corresponds to the optimal selection.

$\theta$	$\scriptstyle \phi _{\kappa }$	$\scriptstyle f_{i}(x)$
$\frac{cos^{-1}\big (\frac{A\cdot B}{\Vert A\Vert \Vert B\Vert }\big )}{\pi }$	$\scriptstyle exp\Big (\frac{-\left\Vert x_{i}-x_{j}\right\Vert ^{2}}{2\sigma ^{2}}\Big )$	$\phi _{S}(rsv\{svd(cor|x_{i},S|^{\xi })\})$
$\int _{-\infty }^{\infty }p_{A}(x)log\big (\frac{p_{A}(x)}{q_{B}(x)}\big )dx$	$\scriptstyle tanh(\frac{1}{N} x_{i}\cdot x_{j}+\xi )$	$\phi _{S}(rsv\{svd(cor|x_{i},S|^{\xi })\})$
$\int _{t_{0}}^{t_{f}}l_{i}(\tau ){\overline{l}}_{j}(\tau )d\tau$	$\scriptstyle 1-\frac{\Vert x_{i}-x_{j}\Vert ^{2}}{\Vert x_{i}-x_{j}\Vert ^{2}+\xi }$	$\phi _{S}(rsv\{svd(cor|x_{i},S|^{\xi })\})$

### IBD progression can be geometrically interpretable as the intersection of manifolds with boundaries

The physical control of certain changes in the nature of the IBD dynamics is a task highly dependent on the data geometry. In many practical cases, as for example in some image-based problems, data explicitly lies on a manifold. In most biological systems the data are not only high-dimensional, but also highly nonlinear. This is the particular scenario described by the UC and CD graphs analysed above. Nevertheless, those graphs are endowed with a true dimension much lower than the number of features. Manifolds, herein noisy Riemannian manifolds with a measure, are topological spaces that allow UC and CD graphs to be described in terms of the simpler local topological properties of Euclidean space. Thereby manifolds provide a natural framework to understand the structure of any very large high-dimensional data in Biology.

Notably, the data geometry of the IBD status can be learnt by means of an on top space whose differential structure can be determined by the expression profiles of the proteins the WGCNA method selected. Each IBD status is abstracted as a Riemannian manifold; and construct the graph Laplacian from the mentioned on top space bringing the gap between the WGCNA candidates and their localisation in the IBD manifold. Finally, the application defined by the graph Laplacian enables to figure out how the system gets cross from one to other status. Thus, we describe the domain of IBD progression as intersection of three manifolds, i.e., $\Omega _{c}$, $\Omega _{q}$ and $\Omega _{a}$ (Fig. S7).

Let define $f:\mathcal {G}\rightarrow \mathbb {R}$ as the estimate function generated by the eigengenes associated with the WGCNA candidate proteins (Fig. S8 and S9) on the UC/CD graph $\mathcal {G}$. Next, for each couple (*i*, *j*) in $\mathcal {G}$ is already known^18^ that we can smoothly construct a functional by:

1 $$\begin{aligned} S(f) = \sum _{i\sim j}(f_{i}-f_{j})^{2}=\frac{1}{2}f^{t}Lf, \end{aligned}$$

Then data derived from $\mathcal {G}$ can be naturally represented on a manifold preserving adjacency by optimising the following problem:

2 $$\begin{aligned} \min _{ij}\sum _{i\sim j} w_{ij}(f_{i}-f{j})^{2}, \end{aligned}$$

the expression 2 is scaled by the edge weight matrix of $\mathcal {G}$, $w_{ij}=e^{-\frac{||x_{i}-x_{j}||^{2}}{h}}$ and $f:\mathcal {G}\rightarrow \mathbb {R}$ as introduced above. From^18^ the associated best solution is provided by:

3 $$\begin{aligned} Lf=\lambda Df. \end{aligned}$$

That is, the UC/CD graph $\mathcal {G}$ can be represented on a manifold $\mathcal {M}^{k}$ via the graph Laplacian eigenmaps. In particular, the eigenmaps of the initially mentioned eigengenes to each WGCNA candidate proteins lay on points nearby the manifold changes its structure, i.e., singularities in the manifolds intersection. Later, the inspection of the graph Laplacian behaviour enables to define a potential that can model the dynamic of IBD status through the manifolds.

Let’s have a look to the construction of the graph Laplacian $L_{n,h}$ along its scalability factor $\frac{1}{\sqrt{h}}$. To this, we use the Gaussian kernel with bandwidth *h* (SI Text) on the 37 candidate proteins (see previous section) data selected in UC (resp. 19 in CD). Yet, we identify the cross from control to active status of the disease with an intersection-type singularity since it can be naturally considered a “phase transition” as described in Belkin et al.^5,18^. Whereas the active-quiescent pass is interpreted as an edge-type singularity since the manifold sharply changes direction (i.e., from disease to control-like status). We are particularly interested in the former scenario, which involves the intersection of the two different manifolds $\Omega _{c}$ and $\Omega _{a}$. Thus, for a given point $x_{1}\in \Omega _{c}$ consider its projection $x_{2}$ onto $\Omega _{a}$ and its nearest neighbour $x_{0}$ in the singularity. If $n_{1}$ and $n_{2}$, are the directions to $x_{0}$ from $x_{1}$ and $x_{2}$ respectively and $D_{1}$ and $D_{2}$ are the corresponding distances calculated as the Kullback–Leibler divergence^19^ between each status. Then from^5^ $L_{h}f(x_{1})$ can be approximated by $\frac{1}{\sqrt{h}}\Phi _{1}(\frac{D_{1}}{\sqrt{h}})\partial n_{1}f(x_{0}) +\frac{1}{\sqrt{h}}\Phi _{2}(\frac{D_{2}}{\sqrt{h}})\partial n_{2}f(x_{0})$. Note that $\Phi _{1}$,$\Phi _{2}$ are scalar functions meeting in form and type singularity. Both functions are explicitly calculated in 4 for the intersection-type singularity. This approximation requires the application of the Theorem 2 described in the pg. 37 of^5^. And it is a previous step to establish the conditions needed to analyse the behaviour of the graph Laplacian near the intersection of the two 8-manifolds $\overline{\Omega }_{c}$ (i.e. the interior of the smooth $\Omega _{c}$ eventually with boundary) and $\overline{\Omega }_{a}$ embedded in ${\mathbb {R}}^{20}$. By replicating the methodology and notation described in^5,18^ to our case, we fix the restricted function $f_{i}:=f|_{\overline{\Omega }_{i}}$ on $\overline{\Omega }_{i}$, $i\in \{c,a\}$ of a continuous function f defined over $\overline{\Omega }=\overline{\Omega }_{c}\cup \overline{\Omega }_{a}$ to be $C^{2}$*-continuous*. And finally we consider two points, $x\in \Omega _{c}$ nearby the intersection and $x_{0}$ being its nearest neighbour in $\Omega _{c}\cap \Omega _{a}$ (i.e. a smooth manifold of dimension $\le 7$) and their projections $x_{1}$ (resp. $x_{2}$) in the tangent space of $\overline{\Omega }_{c}$ at $x_{0}$ (resp. in the tangent space of $\overline{\Omega }_{a}$ at $x_{0}$). Hence, to a proper distance $\Vert x-x_{0}\Vert =r\sqrt{h}$ with a sufficiently small *h*, we may have

4 $$\begin{aligned} L_{h}f(x)&=\frac{1}{\sqrt{h}}\pi ^{\frac{8}{2}}re^{-r^{2}sin^{2}\theta }p(x_{0}) (\partial n_{1}f_{1}(x_{0}) +\nonumber \\&cos\theta \partial n_{2}f_{2}(x_{0}))+o\Big (\frac{1}{\sqrt{h}}\Big ), \end{aligned}$$

where $n_{1}$ and $n_{2}$ are the unit vectors in the direction of $x_{0}-x_{1}$ and $x_{0}-x_{2}$, respectively, and $\theta$ is the angle between $n_{1}$ and $n_{2}$ measured as the disease incidence during the cohorts recruitment (see Table 2). From equation 4, $L_{h}f(x)$ can be implicitly transform into $\frac{1}{\sqrt{h}}Cre^{-r^{2}}$ to a constant *C* depending on the derivatives of *f* and the position of *x* on or nearby the intersection (see^5,18^).

**Figure 5:**
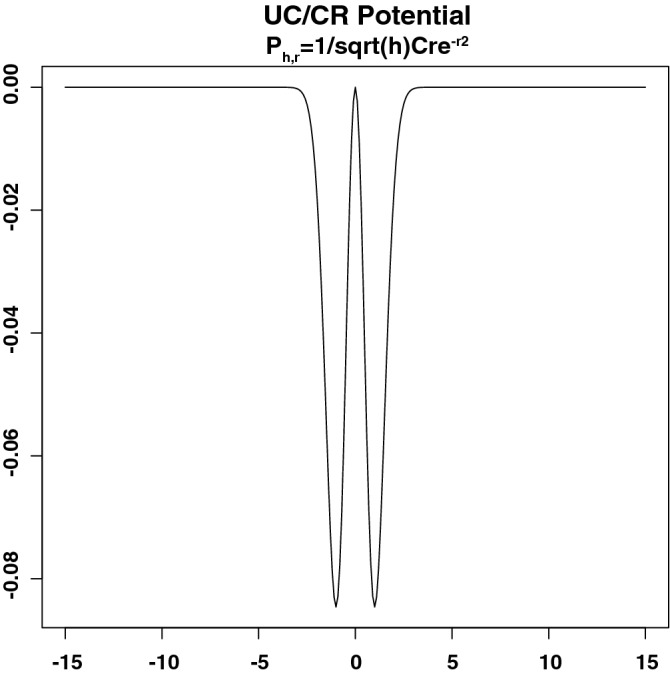
Potential of the UC control-active set. The geometry described, coupled with the abstracted disease-related dynamics through this potential, can be used to prioritise therapeutic interventions.

This result defines a potential (Figs. 5 and S10) properly describing the IBD control-active set, which reinforces the hypothesis exposed in the previous section. There, we claimed how therapeutic targets within the candidate derived from the proteomic coexpression dataset could be effective in the control of certain disease dynamics. The existence of this potential ultimately confirms the hypothesis by simply conducting further dynamical enquiries on it. In the next section, we will describe how we can infer IBD dynamics depending on the positions this potential assigns to each candidate protein in the manifolds. See SI text p 7–10 for further details on the methodological development.

**Figure 6:**
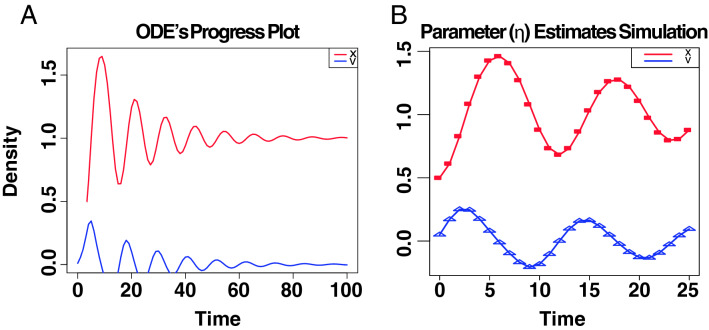
Fitting our model to data. (**A**) Time plot of the ODE’s system associated with IBD status. (**B**) Curves show the model for the best estimated parameters released upon 100 bootstrap iterations; symbols depict the data. Legends amount to the outcome of initial conditions *x* and *v*, which is being simulated over time.

### Therapeutic reconfiguration of the IBD complex space

Finally, we want to identify temporary actions in the expression of the selected therapeutic targets that potentially control the dynamics of IBD status. That would definitely state our initial hypothesis. To this end, we followed Cornelius et al.^8^ in the construction of a control perturbations in an eight-dimensional system. This type of control consists of a particle defined as the image of the eigengenes associated with the selected proteins across patients that are in the kernel of the map $\frac{1}{\sqrt{h}}Cre^{-r^{2}}$. We evaluate, then, their expression in function of this potential that acts as a homeomorphism to assign their corresponding manifold of definition, i.e., $\Omega _{c}$, $\Omega _{q}$ and $\Omega _{a}$. The system of ordinary differential equations (ODEs) describing this particle at or near an intersection-type singularity as introduced above maybe simplified as:

5 $$\begin{aligned} P({{\overline{x}}})&=L_{h}f(x)\nonumber \\ F({{\overline{x}}}, v)&= -dL_{h}f/dx + dissipation, \nonumber \\ dissipation&= -\eta * v \end{aligned}$$

Right-hand side of ODEs defining system, according to Newton’s second law, i.e., if $y = (x, v)$:

6 $$\begin{aligned} dx/dt&= v \nonumber \\ dv/dt&= F(x, v), \nonumber \\&F(x,v)\sim \frac{1}{\sqrt{h}}Cxe^{-x^{2}}-\eta *v \end{aligned}$$

**Figure 7:**
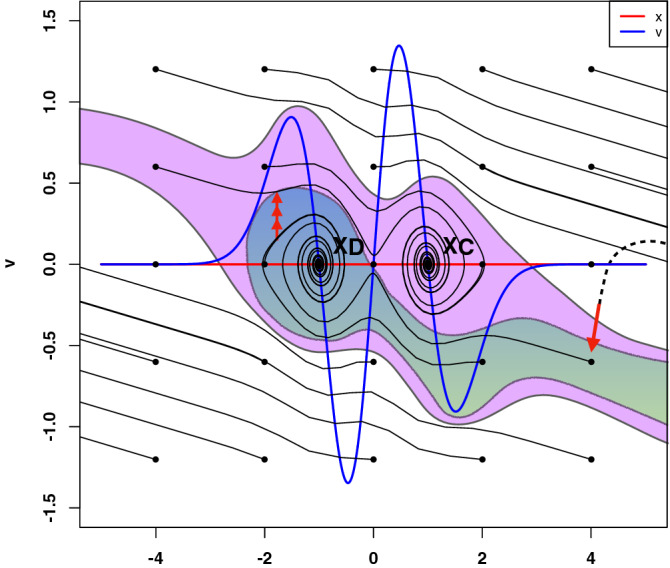
Illustration of the control process in two dimensions. The basins of attraction of the stable states $x_{D}$ (Disease) and $x_{C}$ (Control or Latent) are highlighted in green and violet respectively. White corresponds to unbounded orbits (left and right hand side red arrows). Iterative construction of the perturbation for an initial state in the basin of $x_{D}$ with $x_{C}$ as a target (Left hand side), and for an initial state on the right side of both basins with $x_{D}$ as a target (Right hand side). Dashed and continuous lines indicate the original and controlled orbits, respectively. Red arrows indicate the full compensatory perturbations. Individual iterations of the process are shown in the insets (for clarity, not all iterations are included). Figure adopted from^8^.

Before solving the systems of ordinary differential equations defined in 6, we optimised the dissipation parameter $\eta$ (Fig. 6) by performing 100 bootstrap simulations^20^. If our intuition is correct, we would expect to determine the class of control perturbations that drive a particle near an undesired stable point to an innocuous target state of the disease. In effect, with $\eta =0.1$, the system has two stable fixed points, one at $x < 0$ and the other at $x > 0$ (with $dx/dt = 0$ and $sd\sim O(6.5e^{-8}$)). If we continue the steady state of the system from a starting point near to the origin (i.e. 0.075), there exists a bifurcation in $-0.08$ that well separates the two “basin of attraction” $x_{D}$ and $x_{C}$ representing the bistable domain (Fig. 7) of the IBD status (SI Text). This scenario holds the non-linear dynamics implicit in the progression of IBD. Importantly, it captures the iterative interventions on the expressions of the previously selected proteins required to effectively brings the system to a non-active status from an uncontrolled path of the IBD status. Let $y_{D}$ and $y_{C}$ be the positions of stable fixed points minimising $L_{h}f(x)$ at $x_{D}$ and $x_{C}$ respectively. We first fix an initial state near $x_{D}$ to take then a state in the basin of $y_{D}$, and try to drive it to the basin of $y_{C}$. This resulted in a pertinent class of control perturbations highlighted as red arrows on the left hand side of Fig. 7. Complementary, we also calibrated the class of compensatory perturbations causing the dynamic of a state on an unbounded orbit, i.e. $x \rightarrow +\infty$, be driven onto the basin of $y_{C}$. Similarly to the previous case, this class is also represented by a red arrow, but this time on the right hand side of Fig.  7. Specifically, we find that we are able to rescue the same pre-active or quiescent status above with an average distance in norm of $O(e^{-20})$ of a feasible target status. These interventions affect a small number of proteins that in turn are multi-target, which is highly desirable provided IBD status progression is believed to be in a multi-facet cellular components synchrony. The dynamics of UC and CD share a unique pattern, but involving a few different proteins in the reconfiguration of their systems. Consequently, our initial hypothesis would be proved by programming actions performed by the IBD potential on the promising therapeutic targets within the co-expression dataset.

An experimental validation of this hypothesis by systematic screenings of our proteomic data would be unaffordable, enhancing the potential of our methodology. See SI text p 11–12 for further details on the methodology.

## Conclusions

We introduce a systematic strategy to identify potential immune drivers whose variation in expression can explain the different status displayed in the evolution of two cohorts of IBD patients. To this end, we model IBD status by the intersection of special geometric varieties called manifolds. And leverage the graph Laplacian to identify points, on or nearby their intersection, with highly specific module regulators of protein co-expression graphs. These graphs were constructed based on high-throughput proteome screenings of the two samples of IBD patients, i.e., replica 1 and replica 2 cohorts. Then to make this methodology more biological meaningful, we can test our predictions by means of experiments that study how specific interventions influence the reprogramming of IBD status, either via high-throughput sequencing surveys of validation populations^21^ or by immunofluorescence specific to given antibodies^22^. The comparison between theory and experiment will provide insight into the functional constraints of immune system in the recognition of bio-drivers varying when facing an intestinal chronic inflammatory threat.

The continuous trade-off amongst the available resources in living systems determines, in a certain way, their response to many situations of stress. To put this mechanism of response in motion, organisms tend to deploy a large diversity of components, such as cell types or proteins^23,24^, each sensitive to a small section of their domain. For example, the colon supports the interplay between reactive oxygen and nitrogen species overproduction or cytokines growth factors, that collectively represent a pivotal role of behaviourally aspects in IBD-induced carcinogenesis^25^. Likewise, the role of the binomial composed by the innate and adaptive immune system in IBD therapies involves dozens of feedback loops invoking and sustaining chronic inflammation. However, how the immune system sparks a particular response to repel an IBD threat remains confusing, though it is thought to be immune and non-immune based, with an accepted role of the gut microbiota and non-immune derived cells of the inflammatory cascade including chemokines and inflammasomes^26^. In this case, the multifaceted information process limits the repertoire of the immunological machinery. To deal with those specific environmental forces, living systems wisely prioritise their resources in accordance with their costs, and constraints^27^. In this work, we have shown how the immune system response in IBD is subjected to such combination of elements, what could fix the status of IBD during its evolution. Our finding reproduces an optimal framework to detect novel immune driver-type specific to IBD status and relates it to the concept of their non-linear dynamics nearby singular topological settings^28^. In this context, limiting regions of phenotypic space are modelled by means of Riemannian manifolds revealing themselves suitable to reflect the important competition between resources and costs when that eventually exceeds a vital threshold in IBD. In general this unbalanced response forces the system to drive trajectories of IBD patients to undesirable status of disease^29^, our model would lead those trajectories to a region of initial conditions whose trajectories converge to a desirable status –similar to the “basin of attraction” introduced in^8^. The connection between the identified immune drivers-type specific and their implication in the evolution of IBD network status becomes even clearer analysing the results yielded by our dynamical model where the pass from one to other status could explain the synergy between innate/adaptative immune resources and their energetic cost and grow in relation to their success in securing resources. Although this study is a characterisation by oversimplification of the adaptive immune system detecting dysplastic lesions in IBD, we expect that our methodology and results will be instrumental also for other diseases and thus have a more wider application for the biomedical field and associated health care systems.

## Materials and methods

The calculations related to these sections were implemented using scripts based on R for weighted graphs analysis (wgcna package^30^), in-house Matlab (2011a, The MathWorks Inc., Natick, MA) functions and supported R^31^ for the analysis of singularities on manifolds, and Python for nonlinear optimisation of control perturbations.

### Data

Samples of 30 $\mu$g of protein prepared from five group of patient biopsies from sigmoid colonic inflamed mucosa extracted from two cohorts (CTRL, active Crohn, quiescent Crohn, active UC, quiescent UC; 8 samples by group). See SI text p3–4.

### Proteomic screening

LC-MS/MS acquisition in samples of $30 \mu g$ of 3, 910 proteins was prepared from the groups of patient biopsies running on a NUPAGE 4-12% acrylamide gel (Invitrogen) and stained in Coomassie blue (Simply-blue Safestain, Invitrogen). Peptides and proteins identifications and quantification by LC-MS/MS were implemented by Thermo Scientific, version 2.1 and Matrix Science, version 5.1. SI text p4.

### WGCNA

We adopted the standard flow of WGCNA^11^ to constructing the protein graphs of UC and CD, detecting protein modules in term of IBD status co-expression and detecting associations of modules to phenotype i.e., control, active and quiescent disease with a soft-threshold, $\xi$, determined according to the scale-free topology criterion (SI Text). Gene ontology analyses coupled with bioinformatics approaches revealed drug targets and transcriptional regulators of immune modules predicted to favourably modulate status in IBD. SI text p5.

### Notes on the graph Laplacian limit

The points nearby phenotypic changes of the disease space are not interior though. Thereby, we need to use the definition of limit from^5,32^ if we want to analyse the behaviour of our graphs Laplacian at very large scale. SI text p12.

### Nonlinear optimisation

We learn from^8^ how to optimise the interventions set needed to control the evolution of our disease model. This control procedure is iterative and consists of minimising the residual distance between the target state, $x^{*}$ , and the system path *x*(*t*) at its time of closest approach, $t_{c}$. To ensure the existence of admissible perturbations in the system herein represented by the vector expressions 7,9 and also to limit the magnitude of the solution $\delta x_{0}$ of the optimisation problem 10, some few constraints must be introduced (SI Text). Then finding the particular solution, $\delta x_{0}$, becomes a nonlinear programming problem (NLP) that can then be properly defined as:

7 $${\min}\, |x^{*}-(x(t_{c})+M(x'_{0},t_{c})\cdot \delta x_{0})|$$

8 $$\rm {s.t.} \, g(x_{0},x'_{0}+\delta x_{0})\le 0$$

9 $$\begin{aligned}&h( x_{0},x{^\prime}_{0}+\delta x_{0})=0 \end{aligned}$$

10 $$\begin{aligned}&\epsilon _{0}\le |\delta x_{0}|\le \epsilon _{1} \end{aligned}$$

11 $$\begin{aligned}&&\delta x_{0}\cdot \delta x^{p}_{0}\ge 0 \end{aligned}$$

where the matrix $M(x_{0}; t)$ is the solution of the variation equation $dM=dt = DF(x)\cdot M$ subject to the initial condition $M(x_{0}; t_{0}) = 1$. And $\delta x^{p}_{0}$ denotes the incremental perturbation from the previous iteration. SI text p12.

### Ethics declarations

The protocols involving human participants conformed to the local Ethics Committee (CPP-Île de France IV No. 2009/17) and to the principles set out in the WMA Declaration of Helsinki, and the Belmont Report from the Department of Health and Human Services. Human ascending colon and ileal biopsies were obtained from the IBD Gastroenterology Unit, Beaujon Hospital and a written informed consent was obtained from all the patients before inclusion in the study.

## Supplementary information

Supplementary Information1

## Data Availability

The datasets and code used in this article have been uploaded to Figshare repository and can be found at https://figshare.com/s/5a75fe48258b1f1c4f11, https://doi.org/10.6084/m9.figshare.11672391, and https://doi.org/10.6084/m9.figshare.11672373 after the release date.
