# Supplementary material for: Singular manifolds of proteomic drivers to model the evolution of inflammatory bowel disease status
Source: Sci Rep. 2020 Nov 4;10:19066. doi: 10.1038/s41598-020-76011-7 (PMC7643119; doi:10.1038/s41598-020-76011-7)
Supplement: Supplementary file 1 — Supplementary Information1 [file 41598_2020_76011_MOESM1_ESM.pdf]

Supplementary Material for

Singular manifolds of proteomic drivers to model the evolution of Inflammatory Bowel Disease status

Ian Morilla[1, 2,*], Thibaut Léger[3], Assiya Marah[4], Isabelle Pic[4],

Hatem Zaag[2], and

Eric Ogier-Denis[1]

[1]INSERM, Research Centre of Inflammation, laboratoire d'excellence Inflamex, BP 416, Paris, France

[2]Université Sorbonne Paris Nord, LAGA, CNRS, UMR 7539, laboratoire d'excellence Inflamex, F-93430, Villetaneuse, France

[3] UMR 7592 CNRS, Institut Jacques Monod, Proteomic Service, Université Paris Diderot, Paris, France

[4] Inception IBD, Inc., Montreal, Canada

*To whom correspondence should be addressed. E-mail: morilla@math.univ-paris13.fr

Full Description of Methods 3
Liquid Chromatography with Mass Spectrometry (LC-MS/MS) Acquisition... 3

Peptides and proteins identifications and quantification by LC-MS/MS 4

How to perform the Weighted Gene co-Expression Network Analysis 5

Manifold Assumption of Data Representation in IBD 7

Laplace-Beltrami Operator Basis 7

Data Representation: Graph Laplacian Eigenmaps 8

Singular Manifolds of IBD Status 8

Graph Laplacian of IBD Status 9

Notes on the Limit of Graph Laplacian defined upon Singular Manifolds 10

ODE System for IBD Status Dynamic 11

Optimisation of the Dissipation Parameter 11

Compensatory Perturbations (Nonlinear Optimisation) 12

Supplementary Results 12

Full Description of Methods

Below, we describe the methods and notation used in this work that were borrowed and adapted from previous results introduced by Horvath et al., 2008 (pg 5), Belkin et al., 2012, Belkin et al., 2015 (pgs 7-10), and Cornelius et al., 2013 (pgs 11-12) among others. Intellectually the work was already done independently, then, we only glued the pieces!

Liquid Chromatography with Mass Spectrometry (LC-MS/MS) Acquisition

Samples of 30 µg of protein prepared from five group of patient biopsies (CTRL, active Crohn, quiescent Crohn, active UC, quiescent UC; 8 samples by group) were run on a NUPAGE 4-12% acrylamide gel (Invitrogen) and stained in Coomassie blue (Simply-blue Safestain, Invitrogen). 3 gel plugs by sample were cut for each condition and reduced with 10mM dithiothreitol (DTT), alkylated with 55mM iodoacetamide and incubated with 20 µL of 25 mmol L^{-1} NH_4HCO_3 containing sequencing-grade trypsin (12.5 µg mL^{-1}; Promega) overnight at 37°C. The resulting peptides were sequentially extracted with 30% acetonitrile, 0.1% formic acid and 70% acetonitrile, 0.1% formic acid. Peptides mixtures were pooled by sample and analyzed by an Orbitrap Fusion Tribrid coupled to a Nano-LC Proxeon 1000 equipped with an easy spray ion source (all from Thermo Scientific). Peptides separation performed by chromatography under the following conditions: Acclaim PepMap100 C18 pre-column (2 cm, 75 µm i.d., 3 µm, 100 Å), Pepmap-RSLC Proxeon C18 column (75 cm, 75 µm i.d., 2 µm, 100 Å), 300 nl/min flow rate, using a gradient rising from 95 % solvent A (water, 0.1 % formic acid) to 40 % B (80 % acetonitrile, 0.1% formic acid) in 120 minutes, followed by a column regeneration of 20 min.

Peptides were analyzed in the Orbitrap cell, in MS, at a resolution of 120,000 (at *m/z* 200), with a mass range of *m/z* 350-1550 and an AGC target of 2 x10^5. Fragments were obtained by high collision-induced dissociation (HCD) activation with a collisional energy of 30%. MS/MS data were gathered from the ion trap in the top-speed mode, with a total cycle of 3 seconds, with an AGC target of 5x10^4, a dynamic exclusion of 50 seconds and exclusion duration of 60 seconds. The maximum ion accumulation times were set to 100 ms for MS acquisition and 30 ms for MS/MS acquisition in parallelization mode.

Peptides and proteins identifications and quantification by LC-MS/MS

Identification procedure: The Proteome Discoverer software (Thermo Scientific, version 2.1) and with the Mascot search engine (Matrix Science, version 5.1) were used in the MS and MS/MS data processing. For the precursor ion we set to 7 ppm the mass tolerance maximum threshold and 0.5 Da for fragments. The following variable modifications (2 maximum per peptide) were allowed: carbamidomethylation (Cys), oxidation (Met), phosphorylation (Ser, Thr, Tyr), acetylation (N-term of protein). More than two missed cleavages were not considered for the trypsin protease. The MS/MS identification step performed on the SwissProt database (02/16 version) using the *Homo sapiens* taxonomy as base. Peptide Identifications were validated using a 1% FDR (False Discovery Rate) threshold calculated with the Percolator algorithm. The relative quantification of protein abundances is measured by means of Progenesis QI - Proteomics software (version 4.0, Waters) - using co-detection in order to eliminate missing values. The relative quantitation of proteins according to the five groups (CTRL, active

Crohn, quiescent Crohn, active UC, quiescent UC) was performed using a between subject analysis and a Hi-3 method (for which the three most abundant peptides were used for protein quantification). Abundance variations of proteins with an ANOVA p-value under 0.05 and with at least two identified peptides were further considered.

How to perform the Weighted Gene co-Expression Network Analysis

The WGCNA R package enables the construction of co-expression network using the normalised data of the 40 LC-MS/MS acquired runs in the two replica samples just described in the previous section. We set the threshold power to 10 since it was the supremum in the scale-free R^2 fit of 0.8. Briefly, the network was created calculating topologic overlap (TO) with bicor correlation function, then proteins were hierarchically clustered using 1-TO (dissTOM) as the distance measure. Initial module assignments were determined by using dynamic tree-cutting, using default parameters except deepSplit =0, mergeCutHeight=0.20, minModulesize = 30, pamStage = FALSE, and pamRespectsDendro = TRUE, but proteins were allowed to be reassigned to modules with better correlation if $p < 0.05$ for those correlations. The network type was signed, so that anticorrelated proteins were not assigned to the same module. Resulting 16 (resp. 18 for CD) modules or groups of co-expressed proteins for UC ranging in size from 540 proteins (resp. 305 for CD) (turquoise) to 73 proteins (cyan, resp. 81 in tan for CD) were used to calculate the eigengenes (MEs; or the 1st principal component of the module). MEs were correlated with the status of IBD in terms of biological trait. We capture the individual interaction of genes with the IBD status by defining Protein

Significance PS as the correlation between the protein and the IBD status in absolute value. For any given module, we set the quantitative measure named module membership (MM) as the correlation of the module eigengene and the protein expression profile.

In the validation WGCNA, scale free topology was achieved with beta (power) set at 17.5, and other similar to above function parameters were deepSplit = 4, minModulesize = 3, mergeCutHeight = 0.12, pamStage = TRUE, pamRespectsDentro = TRUE, reassignThresh = 0.05. The WGCNA::modulePreservation() function was used to estimate the preservation rate stats of each module between the discovery and validation cohorts of IBD patients with parameters nPermutations=30, maxGoldModuleSize=100, maxModuleSize=400. Functional analysis of over-represented processes was performed with an in-house tailored script testing for hypergeometric overlap of gene symbol membership in modules across the derivation and validation networks using the fisher.test() function achieveing to a consesus with the resulting scores obtained by means of DAVID, GORILLA, STRING, KEGG and PANTHER data bases.

WGCNA network gene-level kME values (gene expression correlation to the module eigengene for the module to which each gene belonged) were used to rule out the measured gene list to genes with kME > 0.65 and membership in any of 4 modules (resp. 2 in CD) of particular interest in UC. R code is provided online via synapse.org (https://www.synapse.org/XXXXXX).

Manifold Assumption of Data Representation in IBD

In many biological systems data explicitly lies on a manifold. It is also normal that those data exhibit structures of high dimensionality and nonlinearity. Still, they can be described by a number of components much lower than their current features. Manifolds, herein noisy Riemannian manifolds with a measure, provide a natural framework to understand the structure of high-dimensional data in Biology.

We enquire how our data shape might affect that we state about similarity. Thus, we consider a function $f: X \to \mathbb{R}$ that potentially learns the shape of a data set. Then by the manifold assumption stated in Belkin et al., 2012, *we claim the inner product* $\langle f, \Delta_p f \rangle_X$ *is small.* Subsequently, data shape can be closely traced using such function *f*.

Laplace-Beltrami Operator Basis

The Laplace operator acts, in a certain way, as precursor of the basis required for the function used in the geometry of data. The Laplace – Beltrami operator can be inferred by generalisation of Fourier analysis. If we define $f: \mathcal{M}^m \to \mathbb{R}$ and $exp_p: T_p\mathcal{M}^m \to \mathcal{M}^m$ as the application to be estimate defined from the manifold of dimension $m\ to$ $\mathbb{R}$ (as describe in the previous section) and the corresponding application defined from its tangent space respectively. Then, the operator is defined as follows:

$$\Delta_{\mathcal{M}} f(p) = -\sum_i \frac{\partial^2 f(exp_p(x))}{\partial x_i^2}$$

Data Representation: Graph Laplacian Eigenmaps

Consider a Graph G and its set **f** of sub functions f_i defined as indicated in the previous section and this for each $(i,j) couple\ of\ nodes\ in\ the\ graph.$ We smoothly can construct a *functional* by:

$$S(f) = \sum_{i\sim j} (f_i - f_j)^2 = \frac{1}{2} f^t L f$$

Then *data derived from graph can be naturally represented on a manifold* preserving adjacency by optimisation:

$$\min_{ij} \sum_{i\sim j} w_{ij} (f_i - f_j)^2,$$

this is scaled by the edge weight matrix of G, $w_{ij} = e^{-\frac{\|x_i - x_j\|^2}{h}}$ and $f: G \to \mathbb{R}$ is defined as introduce above.

The associated best solution is provided by [3 Belkin Nigoyi 01]:

$$Lf = \lambda D f.$$

Therefore, data derived from the weighted gene co-expression network analysis of IBD can be smoothly represented on a manifold via the graph Laplacian eigenmaps. *But how could this be done attending to the disease status?*

Singular Manifolds of IBD Status

Similarly to Belkin et al. (2012), now we wondered if our system could describe a general model for a singular IBD space based on its status where to learn stratified spaces by means of some submanifolds (control, active and quiescent strata) put together as introduced in Aanjaneya et al. (2011); Bendich et al. (2012); Haro et al. (2008). In that way, we would be able to

reconstruct topological invariants characterising IBD status on manifolds (see, e.g, Chazal et al. (2009); Chazal and Oudot (2008); Niyogi et al. (2008, 2011)). The geometric problem may be briefly described as follows:

Let $\bar{\Omega}$ be the set of *three* Riemannian submanifolds, smoothly selected and being compact, of intrinsic dimension 8 embedded in $\mathbb{R}^{40}$. Among the space of possible singularities, we are basically interested in *two general classes*:

1. **intersection-type (control-active scenario):** That amounts to the in pairwise smooth intersection of submanifolds. The simplest context occurs as such an intersection is done between the interior of the submanifolds, namely: $x \in \Omega_i \cap \Omega_j, i \neq j$;
2. **edge-type (active-quiescent scenario):** Extreme distance points that lie on within the boundaries of two smooth submanifolds. That is $x \in \partial\Omega_i \cap \partial\Omega_j, i \neq j$.

Thus, *the **singular manifold of the IBD status*** can be defined as the union of 3 smooth manifolds $\bar{\Omega}$ endowed with these types of singularities.

Graph Laplacian of IBD Status

Let $X = \{X_1, \cdots, X_{40}\}$ be the set of i.i.d. random samples derived from the two cohorts of IBD patients distribution with density $p(x)$ on $\bar{\Omega}$. Then, X enables to build a weighted graph $G(V, E)$ of each cohort by simply matching the *ith*-sample point X_i with the *ith*-vertex v_i and making correspond a weight w_{ij} to edge e_{ij}. We consider in this study the Gaussian weight function with the Euclidean distance as indicates:

$$w_{40,h}(X_i, X_j) = \frac{1}{20h} K_h(X_i, X_j) = \frac{1}{20} \frac{1}{h^{8/2+1}} e^{-\frac{\|x_i - x_j\|^2_{\mathbb{R}^{20}}}{h}}.$$

Belkin et al., 2012 suggests normalization by $\frac{1}{20}\frac{1}{h^{8/2+1}}$ in order to hold the limit analysis. With the best fitted Gaussian bandwidth of the selected protein candidates as $h = 0.0384$.

Let's define now the edge weight matrix of the graph G as $W_{n,h}$ with $W_{n,h}(i,j) = w_{n,h}(X_i, X_j)$, and $D_{n,h}$ as the diagonal matrix defined by $D_{n,h}(i,i) = \sum_j w_{n,h}(X_i, X_j)$. We may want to introduce the graph Laplacian without normalization scale as the $n \times n$ matrix $L_{n,h} = D_{n,h} - W_{n,h}$. Under all these considerations we can apply the graph Laplacian to any couple of fixed smooth function $f(x)$ and point $x \in \bar{\Omega}$ as follows

$$L_{n,h}\mathrm{f(x)} = \frac{1}{nh}\sum_{j=1}^{n} K_h(x, X_j)[f(x) - f(X_j)].$$

This is the graph Laplacian in the interior points of IBD status manifolds. But more importantly, what would its behaviour be nearby our protein candidates where a phenotypic change produces an intersection-type singularity?

Notes on the Limit of Graph Laplacian defined upon Singular Manifolds

The limit behaviour of graph Laplacians should be observed under a double prism composed by the sample size n and the (Gaussian) kernel bandwidth t. To this end, we first address the limit $h \to 0$ for data at very great scale and later gaining insights onto finite sample and rates as $n \to \infty$ by means of concentration inequalities.

In particular, herein we study how the infinite graph Laplacian $L_h f(x)$acts when x lies on or nearby a singular point with an h small and a fixed function $f : \bar{\Omega} \to \mathbb{R}$.

ODE System for IBD Status Dynamic

The dynamics of a complex network can often be represented by a set of coupled ordinary differential equations. We thus consider a N-node network whose n-dimensional dynamical state x is governed by

$$\frac{dx}{dt} = F(x)\,.$$

In general, the heterogeneity in the physiopathology of IBD progression tends to perturb any of its graphical representation. Let's get this modification underway at a time prior to a given $reference\ t_0$, driving it to a status $x_0 = x(t_0)$ in the attraction basin $\mathcal{B}(x_u)$ of an undesirable phenotype x_u. We seek then to compensate this effect by wisely identifying a perturbation $x_0 \rightarrow x_0'$ to be implemented at time t_0, where x'_0 lies on the basin of attraction $\mathcal{B}(x^*)$ of a desirable status x^*. For clarity, we assume that $x_u\ and\ x^*$ are fixed points, although the same holds to other types of attractors.

Optimisation of the Dissipation Parameter

We fit the dissipation parameters of our IBD model to data (i.e. η). In this analysis both the initial condition, s, and parameters, p, are randomly picked out thanks to a normal distribution. Upon extraction, we fit the model to the data (see Fig. 6B).

Then we calculate the estimated parameters, confidence ranges, and correlations between the parameters. Additionally, the initial condition is read from the data (instead of estimating it).

We also fit simultaneously several data sets by provide a list of data sets to the first data option. When fitting several data sets, some of the parameters could be the same across all data sets, whereas others could differ, and have a unique

value in each data set (see Fig. 6B). Finally, we show how one can bootstrap the data by sampling (with recruitment) from every individual data set, and re-fit the samples using the best parameters as an initial guess (Fig. 6A). These calculations were implemented by tailored in-house functions and the R function derived from C-code GRIND [de Boer, R, 2017].

Compensatory Perturbations (Nonlinear Optimisation)

How to iteratively rectify unwanted paths resulted in those spaces by debugging from small compensations, as shown in Fig.7? Given a dynamical system as introduced above and an initial state x_0 at time t_0, a small compensation δx_0 evolves to $\delta x(t) = M(x_0, t_c) \cdot \delta x_0$ at time t. The matrix $M(x_0, t)$ is the solution of the variational equation ${dM}/{dt} = DF(x) \cdot M$ with the initial constraint $M(x_0, 0) = 1$, that is normally invertible [19]. Fixed the time point t_c corresponding to the corrected orbit straightaway after the path attaining the target x^*, we should be able to solve,

$$\delta x_0 = M^{-1}(x_0, t_c) \cdot \delta x(t_c),$$

identifying the compensation δx_0 regarding the system initial condition x_0 that, in the small compensation sub-space verifying $|\partial x_0| \leq \varepsilon_1$, will approximate $x(t_c) + \delta x(t_c)$ to x^* the most (Fig. 7, left hand side red arrows).

Supplementary Results

Table S1. KEGG

Biological Functions in CR	Counts	Magenta

Regulation of immune response	18	$p=2.7e^{-12}$
Immune response	24	$p=3.7e^{-11}$
Innate immune response	23	$p=1.4e^{-9}$
Leukocyte migration involved in inflammatory response	11	$p=1.5e^{-4}$
Antigen processing and presentation of peptide antigen via MHC class I	7	$p= 6.8e^{-4}$
Inflammatory response	11	$p= 1.3e^{-3}$
Immunoglobulin mediated immune response	3	$p= 2.2e^{-2}$
Adaptive immune response	5	$p= 2.4e^{-2}$
Negative regulation of inflammatory response	4	$p= 4.8e^{-2}$
Innate immune response in mucosa	3	$p= 7.0e^{-2}$
Neutrophil activation involved in immune response	2	$p= 9.6e^{-2}$

Table S2.

**Pathways Participating in UC**	**Counts**	**Green**
hsa04141:Protein processing in endoplasmic reticulum	19	$p=2.5e^{-7}$
hsa03060:Protein export	8	$p=1.2e^{-5}$
hsa05152:Tuberculosis	10	$p=3.9e^{-3}$
hsa05164:Influenza A	10	$p=4.3e^{-3}$
hsa04380:Osteoclast differentiation	9	$p=1.0e^{-2}$
hsa04940:Type I diabetes mellitus	6	$p=2.6e^{-2}$
hsa05321:Inflammatory bowel disease (IBD)	4	$p=2.8e^{-2}$
hsa05416:Viral myocarditis	4	$p=2.8e^{-2}$
hsa05212:Pancreatic cancer	4	$p=2.8e^{-2}$
hsa00220:Arginine biosynthesis	5	$p=3.1e^{-2}$
hsa05145:Toxoplasmosis	5	$p=3.1e^{-2}$
hsa00520:Amino sugar and nucleotide sugar metabolism	4	$p=3.4e^{-2}$
hsa04672:Intestinal immune network for IgA production	4	$p=4.8e^{-2}$
hsa05168:Herpes simplex infection	5	$p=6.3e^{-2}$
hsa04612:Antigen processing and presentation	4	$p=7.2e^{-2}$
hsa05332:Graft-versus-host disease	3	$p=8.4e^{-2}$
hsa05320:Autoimmune thyroid disease	6	$p=9.1e^{-2}$
hsa05330:Allograft rejection	5	$p=9.8e^{-2}$
hsa05140:Leishmaniasis	3	$p=9.9e^{-2}$

Table S3.

**Intersection between the green (UC) and magenta (CD) modules**
O14933, O43852, O60711, O75594, P01767, P02788, P04233, P05109, P06702, P08567, P14625, P14780, P20160, P23284, P28838, P28907, P30533, P31997, P35228, P40261, P41218, P42224, P43405, P49863, P50453, P52790, P59665, P61009, P80188, Q03518, Q15005, Q16666, Q5JTV8, Q6UXH1, Q8WU39, Q92604, Q96F15, Q96KC8, Q9BY50, Q9HCN8, Q9Y385, Q9Y617

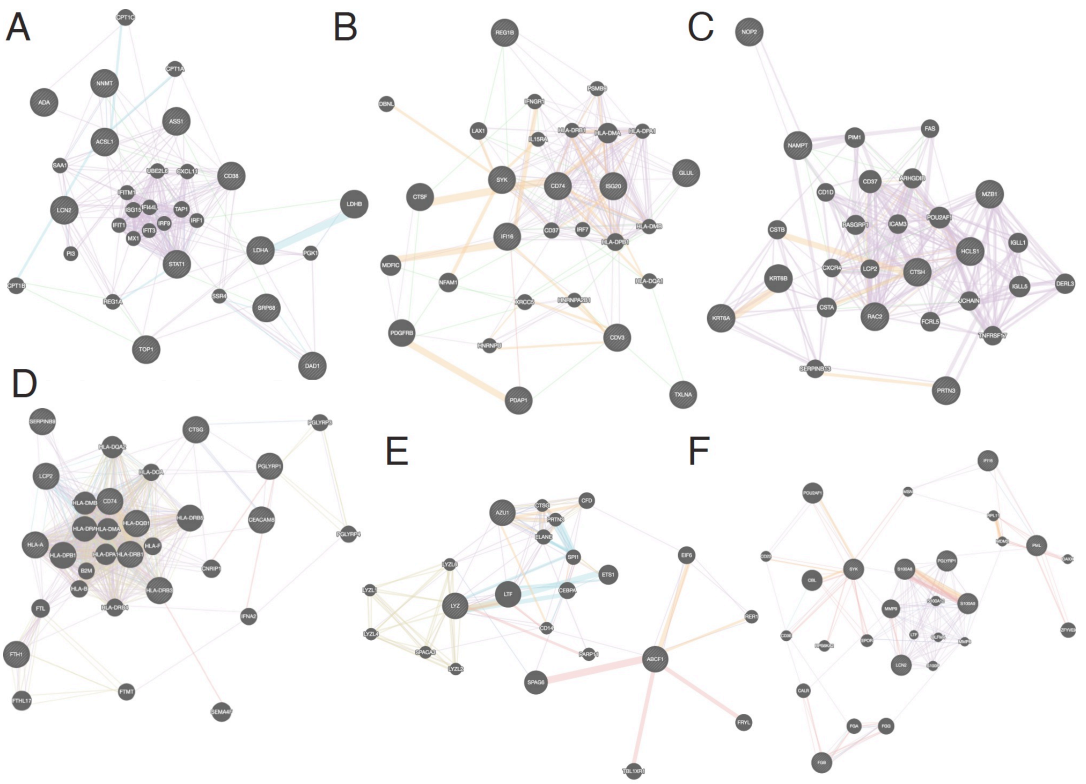

Figure S0. View on detail of the representative repertoire of enriched graphs by functions that are well preserved in UC. (A) Response to drug. **(B)** Cell proliferation. **(C)** Positive regulation of (B)cell proliferation. **(D)** Immune response. **(E)** Inflammatory response. **(F)** Innate immune response. The protein interaction graphs were constructed using Genemania (Warde-Farley et al., 2010). Edge colouring of the graphs amount to: purple stands for Co-expression, orange for Predicted, light-blue for Pathway, light-red for Physical interactions, green for Shared protein domains and blue for Co-localisation.

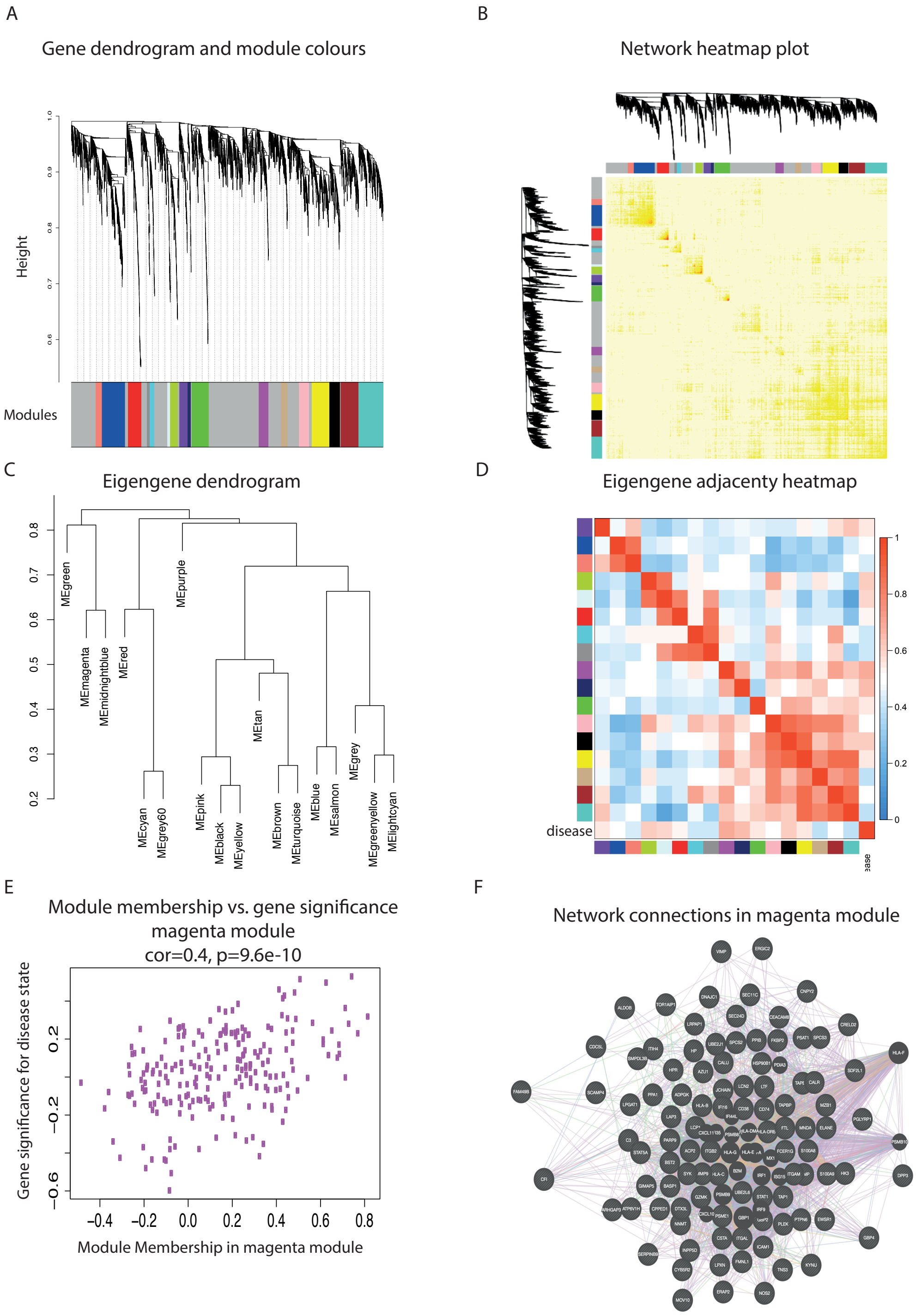

Figure S1. Overview of the protein coexpression network analysis in CR. (A) Hierarchical cluster tree of the 3,910 proteins analysed. The colour strips simply display a comparative overview of module assignments by means of a dynamic method to branch cuttings introduced in (12). Modules in grey are composed by "housekeeping" proteins. **(B)** Topological Overlap Matrix (TOM) plot (also known as connectivity plot) of the network connections. We rank the proteins in the rows and columns following the clustering tree classification. The colour scheme smoothly ranges from faint to thick nuances according to a

low or a higher topological overlap. Typically data clusters along the diagonal. We also include both the cluster tree and module assignment that lie on the left and top sides respectively. **(C)** Hierarchical clustering dendrogram of the eigengenes calculated by the dissimilarity measure $diss(q_1,q_2) = 1-cor(E(q_1),E(q_2))$ (Horvath, 2011). **(D)** Eigengene network visualisation that amounts to the relationships among the modules and the disease status. The eigengene adjacency $A_{q_1,q_2} = 0.5+0.5cor(E(q_1),E(q_2))$ (Horvath, 2011). **(E)** Protein significance versus module membership for disease status related modules. Both measurements keep a high correlation enhancing the strong correlations between the IBD progression and the respective module eigengenes (i.e. greenyellow and green). **(F)** Graph of the green module enriched with subgraphs functionally involved in IBD progression.

A

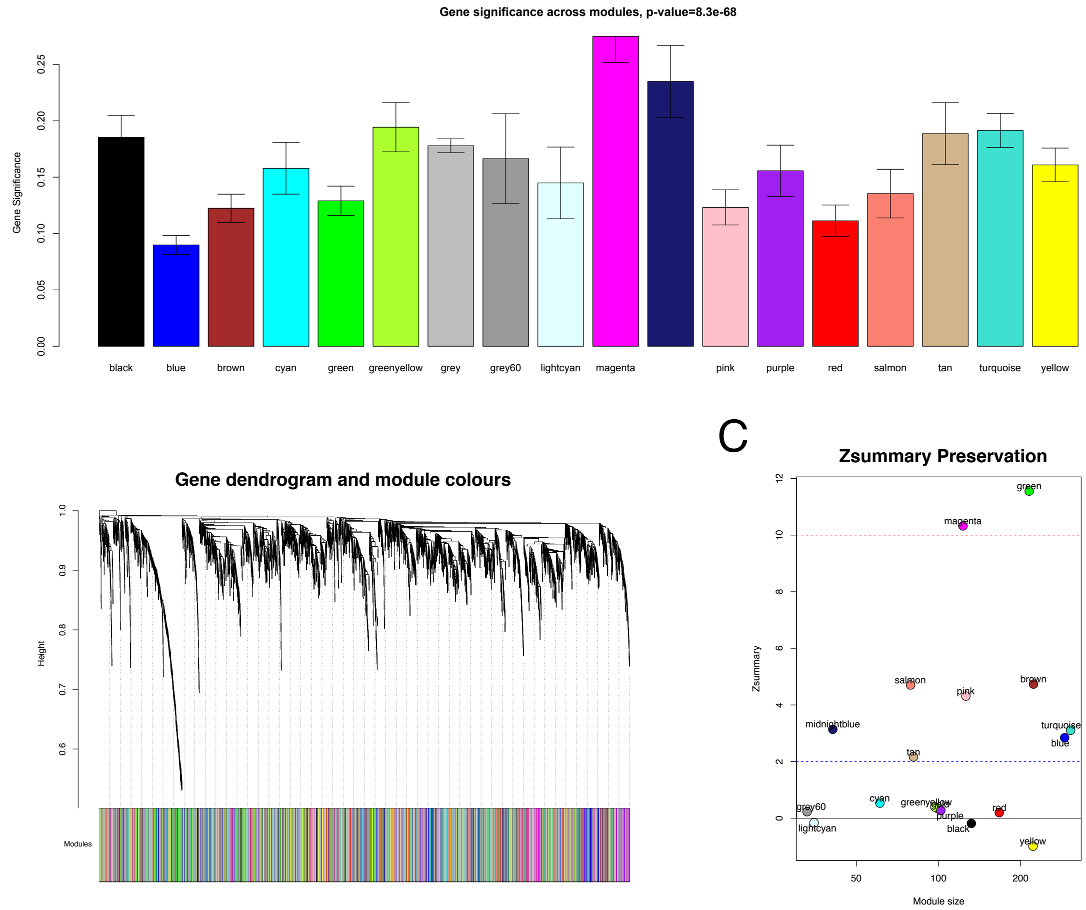

Figure S2. Significance and preservation of CR graph modules. **(A)** The module significance (average protein significance) of the modules. The underlying protein significance is defined with respect to the patient disease status. **(B)** The consensus dendrogram for replica 1 and replica 2 CR co-expression graphs. **(C)** The composite statistic *Zsummary* (Eq.9.1 in (12)). If *Zsummary* > 10 the probability the module is preserved is high (13). If *Zsummary* < 2, we can say nothing about the module preservation. In the light of the Zsummary, it is apparent there is a high correlation with the module size. The green UC module shows high evidence of preservation in its two replica graphs.

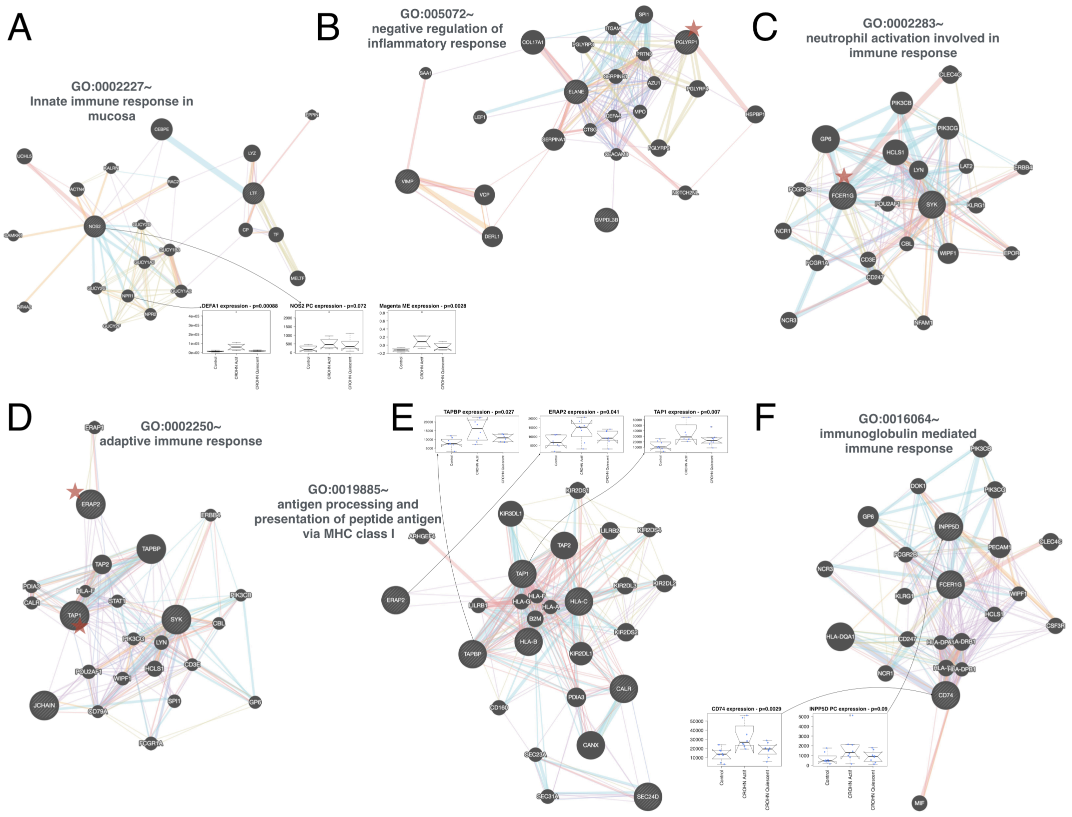

Figure S3. Representative repertoire of enriched graphs by functions that are well preserved in CR. (A) Innate immune response in mucosa. **(B)** Negative regulation of inflammatory response. **(C)** Neutrophil activation involved in immune response. **(D)** Adaptive immune response. **(E)** Antigen processing and presentation of peptide antigen via MHC class I. **(F)** Immunoglobulin mediated immune response. The protein interaction graphs were constructed using Genemania (Warde-Farley et al., 2010). Edge colouring of the graphs amount to: purple stands for Co-expression, orange for Predicted, light-blue for Pathway, light-red for Physical interactions, green for Shared protein domains and blue for Co-localisation. Boxplots of expression for the most attractive drivers respect to UC status are indicated by floating arrows at the top of each subgraph. Initial P upon the boxplot title amount to p-value associated to the IBD status correlation, whereas PC stands for positive control, i.e., proteins already described as IBD related. For the sake of simplicity, we highlighted some candidates simply by its identifiers.

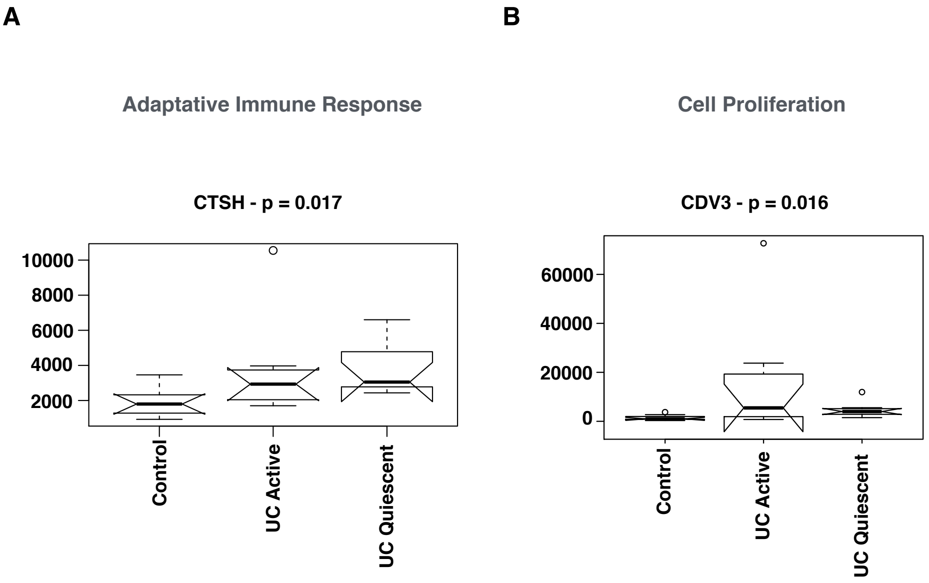

Figure S4. Similar patterns of protein expression in the UC green WGCNA selected module. CTSH and CDV3 barely present the same expression levels between active and quiescent UC status. The units of expression stand for normalised protein abundance.

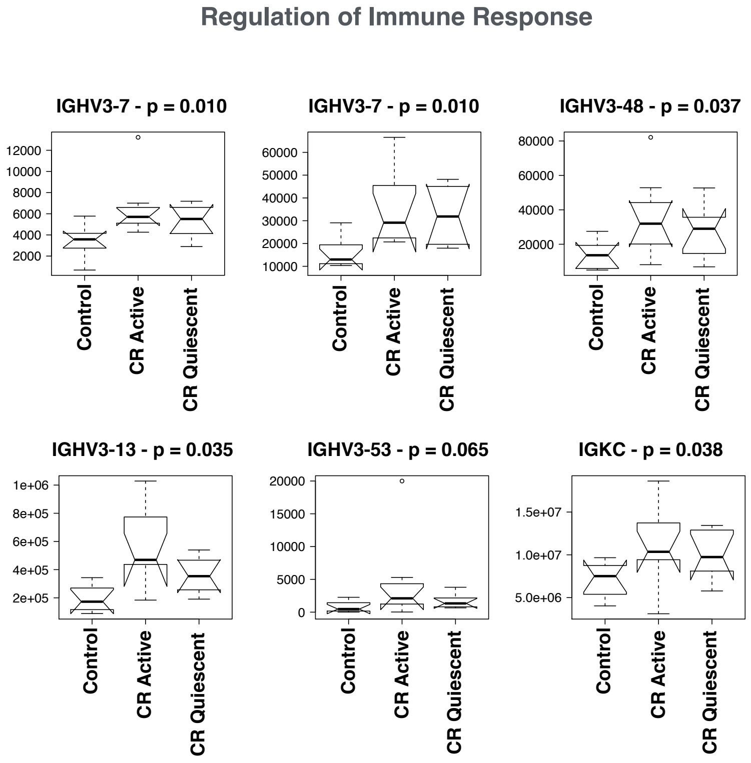

Figure S5. Similar patterns of protein expression in the CR magenta WGCNA selected module. The IGHV3 protein family and IGKC barely present the same expression levels between active and quiescent CR status. The second IGHV3-7 is really IGKV2-30. As earlier, the units of expression stand for normalised protein abundance.

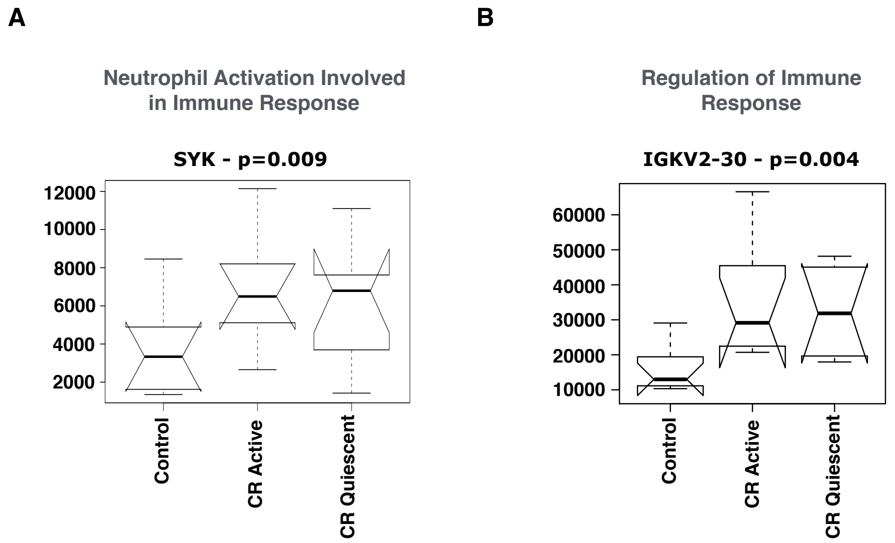

Figure S6. Patterns of protein expression in the CR magenta WGCNA selected module. SYK and IGKV2-30 present themselves more expressed in the quiescent than in the active status during CR progression. Again, the units of expression stand for normalised protein abundance.

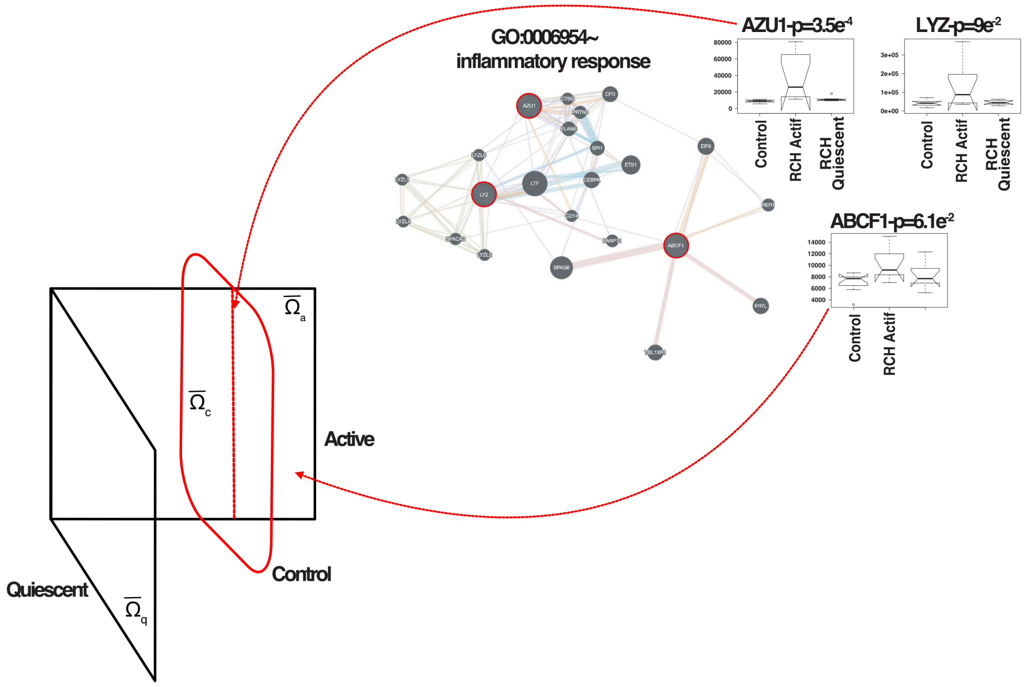

Figure S7. Abstraction of the IBD progression. We describe the domain of IBD progression as intersection of three manifolds eventually with boundaries namely: Ω_c, Ω_q and Ω_a to control, quiescent and active status respectively. The data geometry of IBD status can be learnt through the IBD potential introduced in the main manuscript by using in an on top space the differential structure defined by their WGCNA selected proteins.

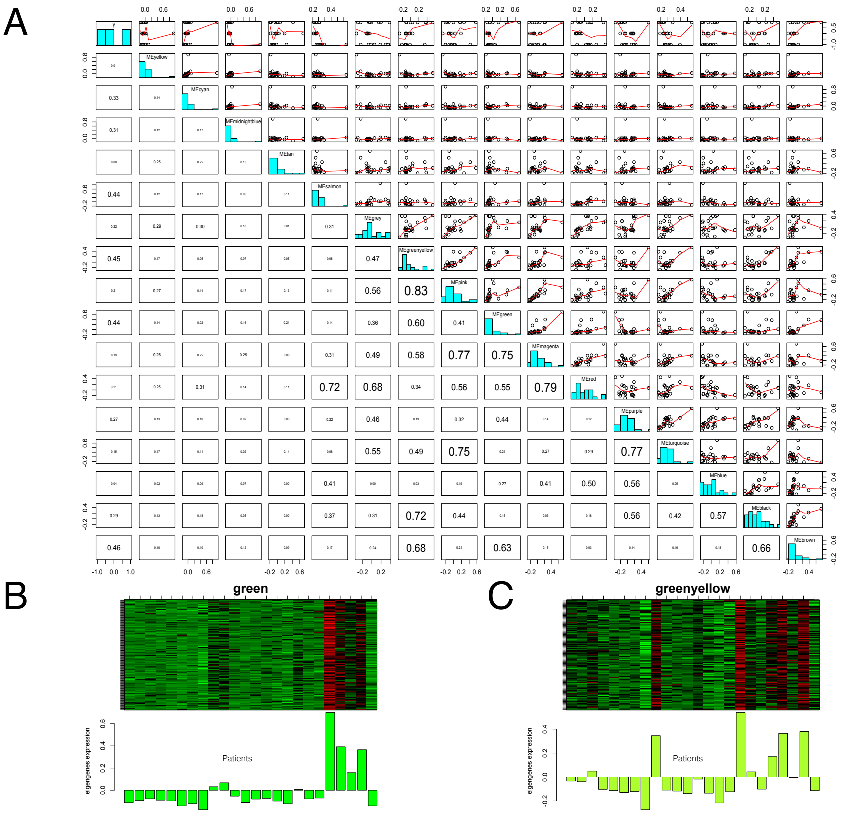

Figure S8. Estimate functions generated by the eigengenes in UC. (A) Dependency boxplots for every correlated module in UC. **(B)** Eigengenes of the green module. **(C)** Eigengenes of the greenyellow module.

A

B

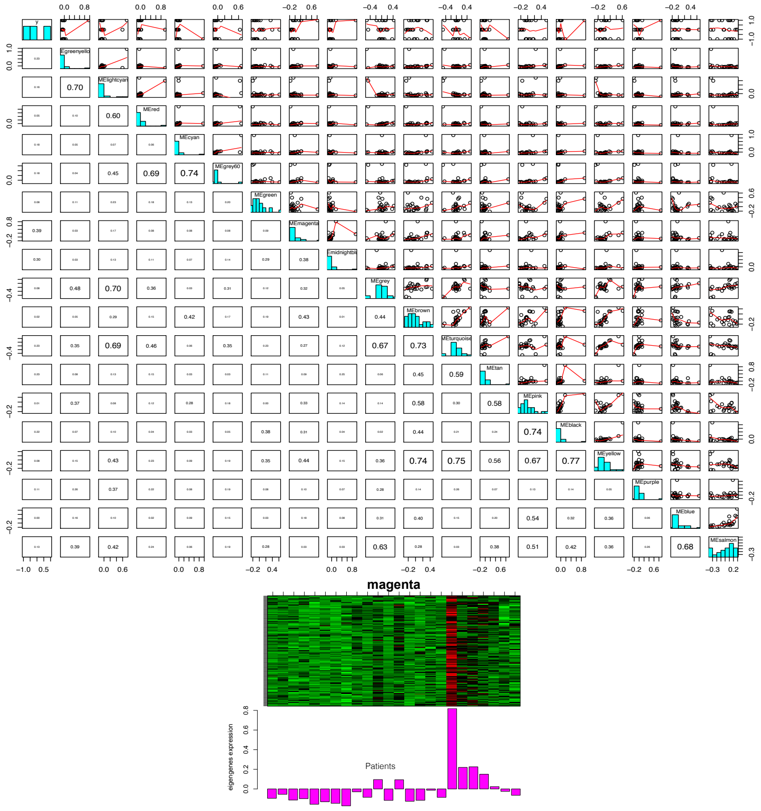

Figure S9. Estimate functions generated by the eigengenes in CR. (A) Dependency boxplots for every correlated module in CR. **(B)** Eigengenes of the magenta module.

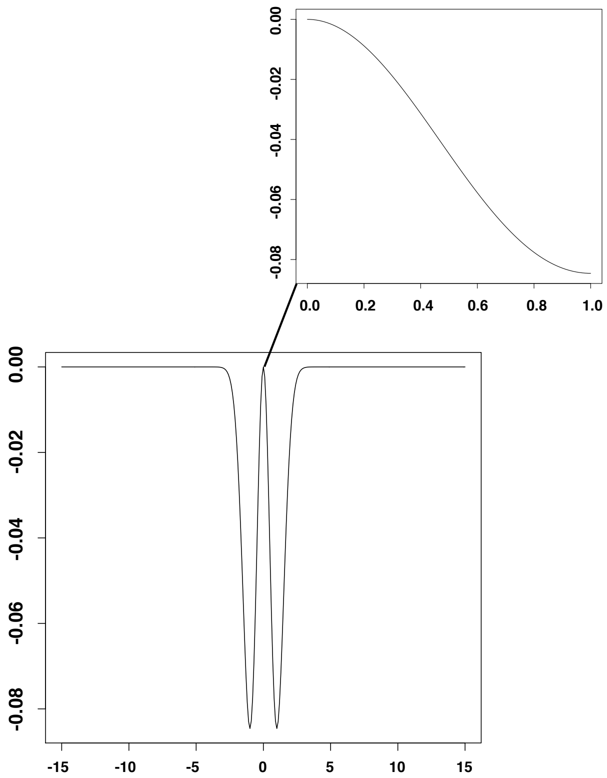

Figure S10. Potential of the UC/CR control-active set. The geometry described in the principal manuscript, coupled with the abstracted disease-related dynamics through this potential defined by the equation $L_h f(x) = \frac{1}{\sqrt{h}} C r e^{-r^2}$, can be used to prioritise therapeutic interventions. Inset: zoom into the positive unitary domain.
